# Supplementary material for: LncRNA SMARCD3-OT1 Promotes Muscle Hypertrophy and Fast-Twitch Fiber Transformation via Enhancing SMARCD3X4 Expression
Source: Int J Mol Sci. 2022 Apr 19;23(9):4510. doi: 10.3390/ijms23094510 (PMC9105468; doi:10.3390/ijms23094510)
Supplement: Supplementary file 1 [file ijms-23-04510-s001.zip › ijms-1637009-supplementary.pdf]

Supplementary Material

Table S1 Sequence of LncRNA *SMARCD3-OT1*

GRCg7b (chr2:118054-118633,133484-133684,134874-135641)

GATGGGAAGGTGAGGTGGTTCACCGGTTGTTGTGGTCTGGGGACCTTTTTCCTGCTTTTCTGGTG
GTGGTCACATCCACCCCTGAAGCTGATCTGATGCTTCCAGATCCCTCTGCGCTGGTGAGAACCCA
GGATCCCTCATCAGAGGTGGACCCTTCCAGCACCATGTCCTGGGATGAATGGTGATTTTGACCAT
CTCCTACCTTGTGCAGACGGACACCCCATGAGGGCCCATATACTCCTCAAACTTTCTTCTGGAAG
ATACCACCATCCAAACCTGCGAGAGTGCATCTGCATTGCTGGCCTTCTTCAGGATGGTGGGGAG
CAGGAGCTGCTTTGATTCTCCTTTCCTCCCCTTTGGGAAGGACACATACCCACCCCAGGAGAGTG
CTCCAGAGGGGATGACATCCCCTTTGCCAGGTCCTGCGGGACAGACTTGCCCAGTTTCCCAGGG
AAGCTGCTGCTCTGTGCTGTCTGCTTGGAAGTGAGACCAGGTCATGTCCAGTGACCAGTCCAAT
CTCAGTCTGTGGTATGGGGCTGTCAGCAGTGCCCTTCTTGGAGAATGACTTCAAAGTATGACGTG
CCTGGAGGAGCCGTGCACCTTCTGAAAAGAACCAGTTTCTTGGTACTGCTTCAGTAGCAATCAA
CACCCAGCTGTTCACCATAGGGACACTGCCATGAGGAGCCAGATCTTTCCAAGCAGGATTCTTG
CCCTTCGGCCCTAGGGAAGGAATTCCCAGCCTGGCCCCGCTCCTTGGGCATCGTGAGGAGCTGG
ATCCAAATGCATCTTCCGCCTGCTCTCTTAGAGCTGCTCCTGCTTCAGGTCAATTAGAATGTCTGC
TGCTCAGTCATCATGATACAATATCTGGTTTCCTACCCCATTCTGCTGTGGATAAGGTTGCTGCTCC
CTCTTGGTCTGCACACATGTGACTGTAGGTGTTTGCCATCTGCCTAGCCTGGCACAGGAGAATGA
AAAGCTGGAAGGCTTGGAAAATGTCCTACCTGCTGCTATAGGCGTATCACAGCTCCTTCAGGACT
GACCTTCCACTGCTGCCTTGTGTTTTTCCAGCCAAAATCTCCTGATACTTTAATGCCCATTTGATTT
TTTTTTTGTCTTTCGACCCAAACGACTGTGAAACTTTCCCCGCGTCTTCTCATGTACATCTCTGAA
GATGGTAAAAAAATGAAAGAAAGAAAGAAACCAGCAAACAGACAAAAACCTGCCTAGAACA
GCCCAGCCATCCCCAGACCTGCTGGCAGCTCCTTGTCTTCCACCACTCGTCATCCTTGTGACATTT
TGTTTACAACCTTTTACTTGCTGTTTGTTTTGTTGGACATTGGGTGTTCACAGTCAGAATTTCAGAC
ATGTTTCCCTGCATTACATTCAAGTTGCTTTTGCCATTTTACTTCTGGCTGCCATTTCTCAAAGCTG
TCGATGTTTGCTGTAATAATGCACTGCCTGAGATGAAAATTTCCTTTTCTCAGGGGTCATCTTTTC
ATAACTTTGTGATTATTTTGCCAAATAAAATGATTTAAAAAGTCA

Table S2 Sequences of different ORFs of LncRNA *SMARCD3-OT1*

ORFs	sequences
**ORF1**	ATGGTGGGGAGCAGGAGCTGCTTTGATTCTCCTTTCCTCCCCTTTGGGAAGGACA CATACCCACCCCAGGAGAGTGCTCCAGAGGGGATGACATCCCCTTTGCCAGGTCC TGCGGGACAGACTTGCCCAGTTTCCCAGGGAAGCTGCTGCTCTGTGCTGTCTGCT TGGAAGTGA
**ORF2**	ATGTTTCCCTGCATTACATTCAAGTTGCTTTTGCCATTTTACTTCTGGCTGCCATTTC TCAAAGCTGTCGATGTTTGCTGTAATAATGCACTGCCTGAGATGAAAATTTCCTTT TCTCAGGGGTCATCTTTTCATAACTTTGTGATTATTTTGCCAAATAAAATGATTTAA
**ORF3**	ATGAATGGTGATTTTGACCATCTCCTACCTTGTGCAGACGGACACCCCATGAGGG CCCATATACTCCTCAAACTTTCTTCTGGAAGATACCACCATCCAAACCTGCGAGA GTGCATCTGCATTGCTGGCCTTCTTCAGGATGGTGGGGAGCAGGAGCTGCTTTGA
**ORF4**	ATGATACAATATCTGGTTTCCTACCCCATTCTGCTGTGGATAAGGTTGCTGCTCCCT

ORFs	sequences
	CTTGGTCTGCACACATGTGACTGTAGGTGTTTGCCATCTGCCTAG
**ORF5**	ATGCTTCCAGATCCCTCTGCGCTGGTGAGAACCCAGGATCCCTCATCAGAGGTGG ACCCTTCCAGCACCATGTCCTGGGATGAATGGTGA
**ORF6**	ATGACTTCAAAGTATGACGTGCCTGGAGGAGCCGTGCACCTTCTGAAAAGAACC AGTTTCTTGGTACTGCTTCAGTAG
**ORF7**	ATGTACATCTCTGAAGATGGTAAAAAAATGAAAGAAAGAAAGAAACCAGCAAA CAGACAAAAACCTGCCTAG
**ORF8**	ATGTCCAGTGACCAGTCCAATCTCAGTCTGTGGTATGGGGCTGTCAGCAGTGCCC TTCTTGGAGAATGA
**ORF9**	ATGCATCTTCCGCCTGCTCTCTTAGAGCTGCTCCTGCTTCAGGTCAATTAG
**ORF10**	ATGAGGAGCCAGATCTTTCCAAGCAGGATTCTTGCCCTTCGGCCCTAG
**ORF11**	ATGGTGATTTTGACCATCTCCTACCTTGTGCAGACGGACACCCCATGA
**ORF12**	ATGGGGCTGTCAGCAGTGCCCTTCTTGGAGAATGACTTCAAAGTATGA
**ORF13**	ATGAAAAGCTGGAAGGCTTGGAAAATGTCCTACCTGCTGCTATAG
**ORF14**	ATGACGTGCCTGGAGGAGCCGTGCACCTTCTGA

Table S3 Sequence of 2,000 bp upstream of *SMARCDC3X4*

CTCTTGCACTTACCTTATGCCTCTTAACAAAGAAAACTCCTTTGCAAACTTCTTTTGTGTAAGAG
TAATGTTGTAGGAGCTTTTTCATACGTTTCCTCTACAGAAAATCTTATCTAAGGACTAGGAAGAG
ATCACTTCCTGTGTGAAACTGCTCAGAGACCTGGAGATTACTGGGCAACATGGGGCCATTTACC
CCAGTCTGTGGTGAAGAAATGATGCATGAGGTGACGATCTCAGGCTCTGCTAAAAGGGCTAAC
TTGGTATAATTGGTTTCTTTCTGCTTCTGATGAAGGACTCCAACATTCTCAAGTTTTCAGATTTCT
GCTTGCTTGTATGTGCACACACACATAAAGAGGGAGTACAAACACAGGCTTTTAACTGGACGT
GACAACAGTGAGAACTGTTACTCTTTACATCTTTCTTATGTTTATGTAGGAGTTTGAGGATCTGC
TGCCAGTTAAAATGTCAAAGTGTCAATGAGAAATCCTTAATATTGGAGAAATCTGGCTTCTGTG
CTTGGTCCTTTGATGTATACTTTGCTATTATTATTAGTTACAATCAATGAACAAAAATTTACAGCT
GGATCATGAGCTTGGAAGCTGAGAGGAGACTGGAAGAAGCTGTGCCAACAGCCAGCCTTTCTC
TATACAAACATTAAAAATACTTGACAAGATGAACAAGCTGCAGTTCACCGGCTCAATGAAGGA
GGTTTTGTTTTGGGGTTAAAGTGTCAGAGCCCAGCAGACTGTCAAGAAAGAACAACATAAAGA
AGTGGAGTGCACGGAGAGCAGAAAGAAGAAAAAAATGTGAAAGAGGAAAGGAAGGCCTAGA
ATTATTTCAGCAACTCAGTGAAATTGGAAGCAGAAGTAGCAACTTTAATCTGAGAAGAAAGCA
AACAGCCTGTATAAAAAAAAAAAAAAAAATCACAAATGTCTACAAAGATAGGCCTGGAATAGA
GAAGCTCTACCTGGATGGTTATCTAGGAAGTCTCAGCAGATGAGAAAAAAATTAATTTTGATTT
TGAAAAAAAAATACATCTACACACTTCCTGATGGAGGGAAAAGCAAGTAGCAATAATAGCAA
TCATATCTTGTGGTAATAACAAATCCCATGCATGTTACCCCTTTCCTCTTGGAGCAGCAAAAGAC
ATGCTGTTTTTCCAGGAATCTGAGAGGAGAAATGCACATTTACTGTTCAGTTTCCTGTTACTTCTT
CACTTTTCTCAGAGGAAAAAGCCATTGGTGAGTACAGTGCAAAGAATGTGGATAAATACAGAA
AGAGTTTAGGATGATCATAGGTAGTTCTCTGCAAGGGGAGCTGCAAGGTAGAAGTTACACTAA
TTTAGATAGAGTAAATCTGTTATAAAAACTGTGGTAGGGTTCAGCTCGAACTTAAAACACAGTC
AAGGCAGAGCTAATGAGAGCAGCCAAATTTTAAGTCATGACACAGTTGAGCAGCTGAACATAA
CTACTTCAGGTTAAGGTACTTTGCTGTCCGGACTCCACCGACCAGACCGCAGGCTTAGACACCT

GGTTCATGTATGTCTACACTTTTTTTTGTCTTCATAAGCAGCCACTTAGGCTATATGTCTTCTGAA
ATTTAAGATTGATCATTGCCCACTATTTTGGTCTTCCTTCCCCCAACACAGCATACTAGTCACTGT
CATTATTGTACTGATGGGTCTTCGTGACTCACCAGCCTCACATGTAGCTTCCACATCCTGCCCCTC
GGGCCTAGTGGCTCAATATAAACTCGGGCCTGGGCATGTGGCTTCTGTACAGGTTATATTGCTG
GTGTGACCGTTTCCTACTTGGCTATAATTATACTTTGCTGTGGGGCCTGTTGTTCCTGTACAGTAG
ACTGACTTTGATGTTTGACTTTAGATGTGCCTCATAACCATGAGCTTTTCTGACGAGTTGGACAC
TGAGCACGAGTACCACCTCCTGGTTTGCGTTGCTTGCCTTATTCAGAAACTGCTGGGCTGGGTCT
TGGTTGGGGAGCC

Table S4 Primers used for qRT-PCR

Gene name	Primer sequences (5'-3')	Size(bp)	Annealing temperature (℃)
SMARCD3-OT1	F: CAGTCTGTGGTATGGGGCT<br>R: AGGCGGAAGATGCATTTGG	276	58
SMARCD3X4	F: CAGTTCCTCTCTCAGCAC<br>R: CAAAATCACCATTCATCC	272	58
GAPDH	F: CAACTTTGGCATTGTGGAGG<br>R: CGCTGGGATGATGTTCTGG	130	58
CDKN1A	F: CGTCTCGGTCTCGAAGTTGA<br>R: CGTCTCGGTCTCGAAGTTGA	133	58
CDKN1B	F: TCGCTGTGCTGGGCTGAA<br>R: CAAGGACGAAAGGATGTGGG	188	58
CCNA1	F: CCCGTAGACCACGAGCAGAT<br>R: TCAGGCAAGGCACAATCTCA	217	58
CCND1	F: CAGAAGTGCGAAGAGGAAGT<br>R: CTGATGGAGTTGTCGGTGTA	158	58
CCNE1	F: CTGCTGGTTCTAACTCCTGCTC<br>R: TGGCGTACTCGATCCATTTCTAT	260	58
MYF5	F: CCTCATGTGGGCTTGCAAA<br>R: CCTTCCGCCGGTCCAT	168	58
MYOD	F: CCTCATGTGGGCTTGCAAA<br>R: CTGGGCTCCACTGTCACTCA	200	58
MYOG	F: CGGAGGCTGAAGAAGGTGAA<br>R: CGGTCCTCTGCCTGGTCAT	320	58
MyHC	F: CTCCTCACGCTTTGGTAA<br>R: CTCCTCACGCTTTGGTAA	213	58
U6	F: CTCGCTTCGGCAGCACA<br>R: AACGCTTCACGAATTTGCGT	189	58
Beta-actin	F: GATATTGCTGCGCTCGTTG<br>R: TTCAGGGTCAGGATACCTCTTT	194	58
TNNC1	F: GTTGAGCAGTTGACAGAAGA<br>R: GAACCATCATAACAAGGAAC	223	58

Gene name	Primer sequences (5'-3')	Size(bp)	Annealing temperature (℃)
TNNI1	F: GAGGAGTGGGAGCAGGAGAT<br>R: TTCGTCCACAATCTCAACCT	231	58
TNNT1	F: GATATTGCTGCGCTCGTTG<br>R: TTCAGGGTCAGGATACCTCTTT	139	58
SRL	F: CCTCCTCGGGCTGGATGACA<br>R: GTTCTTGCTGCTTGCGGTTT	272	58
TNNC2	F: GAGCAGCAAAGATGGCGTCA<br>R: ATCACCGTGCCCAACTCCTT	151	58
TNNT3	F: AGAGGGAAGAAGCAAACAGC<br>R: GTCCCACAGTTCCTTAGCCT	123	58

Table S5 Primers used for RACE

Primer name	Primer sequences (5'-3')	Annealing temperature (℃)
SMARCD3-OT1 5'RACE outer	CTCACTTCCAAGCAGACAGCACAGAGCA	Step down
SMARCD3-OT1 5'RACE inner	GACAGCACAGAGCAGCAGCTTC	Step down
SMARCD3-OT1 3'RACE outer	CCAGGAGAGTGCTCCAGAGGGGATGACA	Step down
SMARCD3-OT1 3'RACE inner	CCAAATGCATCTTCCGCCTGCTC	Step down

Table S6 Primers used for plasmid construction

Primer name	Primer sequences (5'-3')	Size(bp)	Annealing temperature (℃)
SMARCD3-OT1 ORF1	F: ATGAATGGTGATTTTGACCATCTC<br>R: GGGAGCAGGAGCTGCTTTGG	165	58
SMARCD3-OT1 ORF2	F: ATGACTTCAAAGTATGACGTGCC<br>R: AGTTTCTTGGTACTGCTTCAGTGG	78	58
SMARCD3-OT1 ORF3	F: CGTCTCGGTCTCGAAGTTGA<br>R: TGCTCCTGCTTCAGGTCAATTGG	51	58
SMARCD3-OT1 ORF4	F: AATGATACAATATCTGGTTTCCT<br>R: TAGGTGTTTGCCATCTGCCTGG	102	58
SMARCD3-OT1 ORF5	F: ATGTACATCTCTGAAGATG<br>R: AAACAGACAAAAACCTGCCTGG	72	58
SMARCD3-OT1 ORF6	F: ATGTTTGCTGTAATAAT<br>R: ATAATGCACTGCCTGAGATGG	33	58
	F: ATGGTGATTTTGACCATCT	48	58

Primer name	Primer sequences (5'-3')	Size(bp)	Annealing temperature (°C)
SMARCD3-OT1 ORF7	R: TGTGCAGACGGACACCCCATGG		
SMARCD3-OT1 ORF8	F: ATGGTGGGGAGCAGGAGCTGCTT<br>R: TCTGTGCTGTCTGCTTGGAAGTGG	174	58
SMARCD3-OT1 ORF9	F: ATGTCCAGTGACCAGTCCAAT<br>R: AGCAGTGCCCTTCTTGGAGAATGG	69	58
SMARCD3-OT1 ORF10	F: CGGAGGCTGAAGAAGGTGAA<br>R: GGATTCTTGCCCTTCGGCCCTGG	48	58
SMARCD3-OT1 ORF11	F: CTCCTCACGCTTTGGTAA<br>R: TGTCCTGGGATGAATGGTGG	90	58
SMARCD3-OT1 ORF12	F: ATGGGGCTGTCAGCAGTGC<br>R: TGGAGAATGACTTCAAAGTATGG	48	58
SMARCD3-OT1 ORF13	F: ATGAAAAGCTGGAAGGCTTG<br>R: AAAATGTCCTACCTGCTGCTATGG	45	58
SMARCD3-OT1 ORF14	F: ATGTTTCCCTGCATTACATTCAAGT<br>R: ATTTTGCCAAATAAAATGATTTGG	171	58
pEGFP-N1 mutation	F: CACCGGTCGCCACCATTGTTAGCAAG<br>R: CTTGCTAACAATGGTGGCGACCGGTG	4733	60

Table S7 Oligonucleotide used for interference

Fragment name	Sequences (5'-3')
ASO-SMARCD3-OT1	AAACCTGCCTAGAACAGCCC
ASO-SMARCD3X4	GGGAAAGACCAGCACAAAT

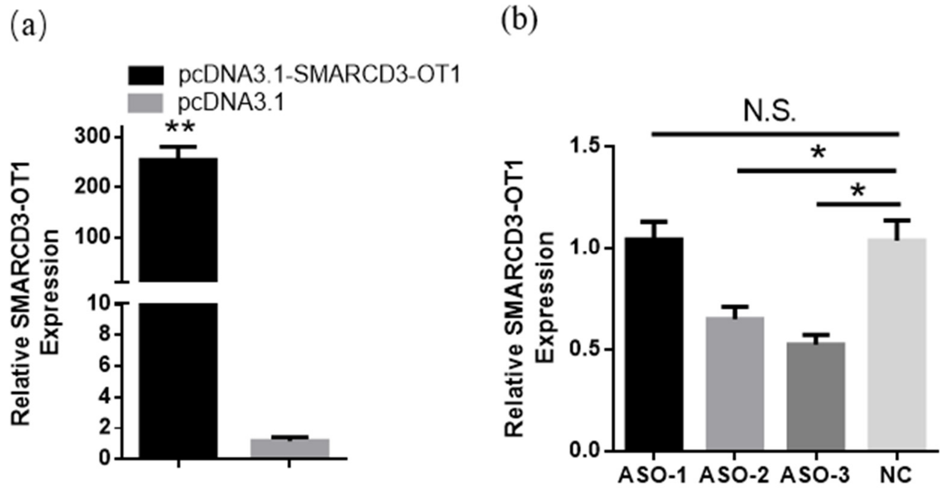

Figure S1 The fold-changes of lncRNA *SMARCD3-OT1* after overexpression and knockdown. (**a**) Relative *SMARCD3-OT1* expression after introducing overexpression vector of *SMARCD3-OT1* into CPMs (n = 6). (**b**) Relative *SMARCD3-OT1* expression after introducing ASO of *SMARCD3-OT1* into CPMs (n = 6). Data are expressed as a fold change relative to the control. Results are shown as mean ± SEM. Statistical significances of differences between means were assessed using an independent sample t-test. *$p < 0.05$, **$p < 0.01$.

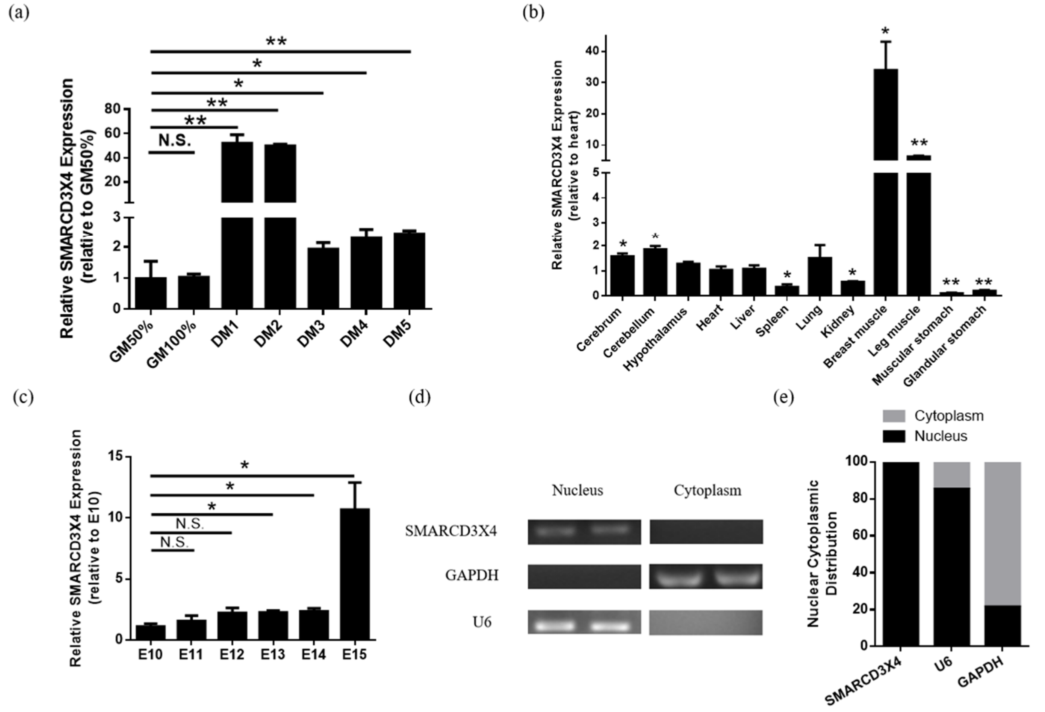

Figure S2 The expression pattern of *SMARCD3X4*. (**a**) Expression levels of *SMARCD3X4* in CPMs cultured in growth medium at 50% and 100% cell confluency (50%GM and 100%GM) and differentiation medium from 1 to 5 days (DM1 to DM5) (n = 6). (**b**) Tissue expression profiles of *SMARCD3X4* in 7-week-old chickens (n = 6). (**c**) Expression levels of *SMARCD3X4* from embryonic day 10 (E10) to E15 (n = 6). (**d, e**) Distribution of *SMARCD3X4* in the cytoplasm and nuclei of CPMs was determined by PCR and qRT-PCR. Data are presented as mean ± SEM. Statistical significance of differences between means was assessed using an independent sample t-test. *$p < 0.05$, **$p < 0.01$.

(a)

TF	Pattern name	Source	Sequence name	Start	Stop	Strand	Score	P value	Q value	Matched motif
SP2		database	SMARCD3X4	378	392	-	18.3966	2.29e-07	0.000764	CCCCCGCCCCCGCCT
SP2		database	SMARCD3X4	726	734	-	13.6735	1.11e-05	0.0285	AGGCGGGGC
SP2		database	SMARCD3X4	372	386	-	13.6379	1.2e-05	0.0135	CCCCCGCCTCCAGGA
SP2		database	SMARCD3X4	28	42	-	13.6207	1.21e-05	0.0135	CCCCCGCCCCAGGAC
SP2		database	SMARCD3X4	760	776	+	13.2921	1.31e-05	0.00998	GCTGGGGCCGGAGCTGG
SP2		database	SMARCD3X4	688	704	-	13.2247	1.39e-05	0.00998	GCGGCGGGCGGAGGCGC
SP2		database	SMARCD3X4	205	221	+	12.809	1.94e-05	0.00998	AGGGGCGGCGGTGCGGA
SP2		database	SMARCD3X4	692	708	-	12.7079	2.09e-05	0.00998	GGTGGCGGCGGGCGGAG
SP2		database	SMARCD3X4	380	396	+	12.6966	2.11e-05	0.00998	GCGGGGGCGGGGGCCGT

(b)

Protein:
MVNILAADWPQPLQAGQGLGRRPPRGRVAQGGARAAPPEEDGAVRGSEPPPPPPRPVSGG
RRFRGPGGGGPGASSRLPGESGRCRRAGGEGGEVDPGTWRGCPGWGLCGWSPEAFLGPRV
PRELRFAPLRVPGVGRAGNTRSRLSDSMAATAAVSPSEYLQPAASTAQDSQPSPLALLAA
TCSKIGPPAVEAAVTPPAPPQPTPRKLVPIKPAPLPLGSGKNSFGILSSKGNLFQIQGSQ
VGTSYPGGQLVFAIQNPTVISKGTRSSANIQYQAVPQIQAAGGQTIQVQPNLTNQIQIIP
GTNQAILTPSSSSHKPVPIKPAPAQKSGASPVQGSSNVVKLTGGSNVTLTLPMNNLVNTT
ESSTQAQVIAESPSKPGKKTRKKAMSPSQPSTVAVAEQVETVLIETTAENIIQAGNNLLI
VQSPGSGQPAVVQQVQVVQPKQESQVVQIPQQALRVVQAASATLPTVPQKSSQNIQIQTT
ESTPTQVYFKTPSGELQTVLLQEAPAVTVAPSSTSCSSPVSRNSGTVTSSKKPTARKERP
LPKIAPAGGVISLSAAQLAAAAQAMQTININGVQVQGVPVTITNAGGQQQLTVQNVSGNN
LTISGLSPTQIQLQMEQALSGEMQPGEKRRRMACTCPNCKDGEKRPGDQGKKKHICHIPE
CGRTFRKTSLLRAHVRLHTGERPFVCNWVFCGKRFTRSDELQRHARTHTGDKRFECAQCQ
KRFMRSDHLTKHYKTHLITKNL

RNA:
CCACGUGUGGGCUGCAGAAGCCGUCGGGAGCCCUUCCUUCUGCGUGGGCUGAGCUUCCCG
GGGGCGUGGAGCGCUCGUCUGACGGCUCCUGGAGGGAACGUCCCCCGUCUCCUCUCCCUG
AGGCACCCGGUGCUGCCCCGCAGUCGGCUCCGGGCUGUCUCCGGCGCUGCCUUUGGACUG
CACCCGGCUGUGUUCUCAGAGGGAGACGCAUCGCCCCGGGUGCUCACUGAGUACAGAGUG
CUCCGCAUCUGACCGCUGCUCUCAGCGCAGGCCGUGCCGCGCUGCUUUGGCAGACGGUUC
UCCCUCCCUCUCUCCCGUUCUUUGCAUCCCGAGCUCUCUCCUCCAGUUCCUCUCUCAGCA
CCACCUGUGGAGGGACCCUGGGGUCAAGCUGCUGGGAAAGACCAGCACAAAUUAAAUGUG
AGAGUGAUGGGAAGGUGAGGUGGUUCACCGGUUGUUGUGGUCUGGGGACCUUUUUCCUGC
UUUUCUGGUGGUGGUCACAUCCACCCCUGAAGCUGAUCUGAUGCUUCCAGAUCCCUCUGC
GCUGGUGAGAACCCAGGAUCCCUCAUCAGAGGUGGACCCUUCCAGCACCAUGUCCUGGGA
UGAAUGGUGAUUUUGACCAUCUCCUACCUUGUGCAGACGGACACCCCAUGAGGGCCCAUA
UACUCCUCAAACUUUCUUCUGGAAGAUACCACCAUCCAAACCUGCGAGAGUGCAUCUGCA
UUGCUGGCCUUCUUCAGGAUGGUGGGGAGCAGGAGCUGCUUUGAUUCUCCUUUCCUCCCC
UUUGGGAAGGACACAUACCCACCCCAGGAGAGUGCUCCAGAGGGGAUGACAUCCCCUUUG
CCAGGUCCUGCGGGACAGACUUGCCCAGUUUCCCAGGGAAGCUGCUGCUCUGUGCUGUCU
GCUUGGAAGUGAGACCAGGUCAUGUCCAGUGACCAGUCCAAUCUCAGUCUGUGGUAUGGG
GCUGUCAGCAGUGCCCUUCUUGGAGAAUGACUUCAAAGUAUGACGUGCCUGGAGGAGCCG
UGCACCUUCUGAAAAGAACCAGUUUCUUGGUACUGCUUCAGUAGCAAUCAACACCCAGCU
GUUCACCAUAGGGACACUGCCAUGAGGAGCCAGAUCUUUCCAAGCAGGAUUCUUGCCCUU
CGGCCCUAGGGAAGGAAUUCCCAGCCUGGCCCCGCUCCUUGGGCAUCGUGAGGAGCUGGA
UCCAAAUGCAUCUUCCGCCUGCUCUCUUAGAGCUGCUCCUGCUUCAGGUCAAUUAGAAUG
UCUGCUGCUCAGUCAUCAUGAUACAAUAUCUGGUUUCCUACCCCAUUCUGCUGUGGAUAA
GGUUGCUGCUCCCUCUUGGUCUGCACACAUGUGACUGUAGGUGUUUGCCAUCUGCCUAGC
CUGGCACAGGAGAAUGAAAAGCUGGAAGGCUUGGAAAAUGUCCUACCUGCUGCUAUAGGC
GUAUCACAGCUCCUUCAGGACUGACCUUCCACUGCUGCCUUGUGUUUUUCCAGCCAAAAU
CUCCUGAUACUUUAAUGCCCAUUUGAUUUUUUUUUUGUCUUUCGACCCAAACGACUGUGA
AACUUUCCCCGCGUCUUCUCAUGUACAUCUCUGAAGAUGGUAAAAAAAUGAAAGAAAGAA
AGAAACCAGCAAACAGAC

Interaction probabilities

Prediction using RF classifier 0.7
Prediction using SVM classifier 0.65

Figure S3 Prediction results of the htTFtaget database and RIPseq website. (a) The SP2 transcriptional factor may combine with the 2000 bp upstream of the functional gene *SMARCD3X4*. (b) The SP2 transcriptional factor is predicted to be combined with *SMARCD3-OT1*.

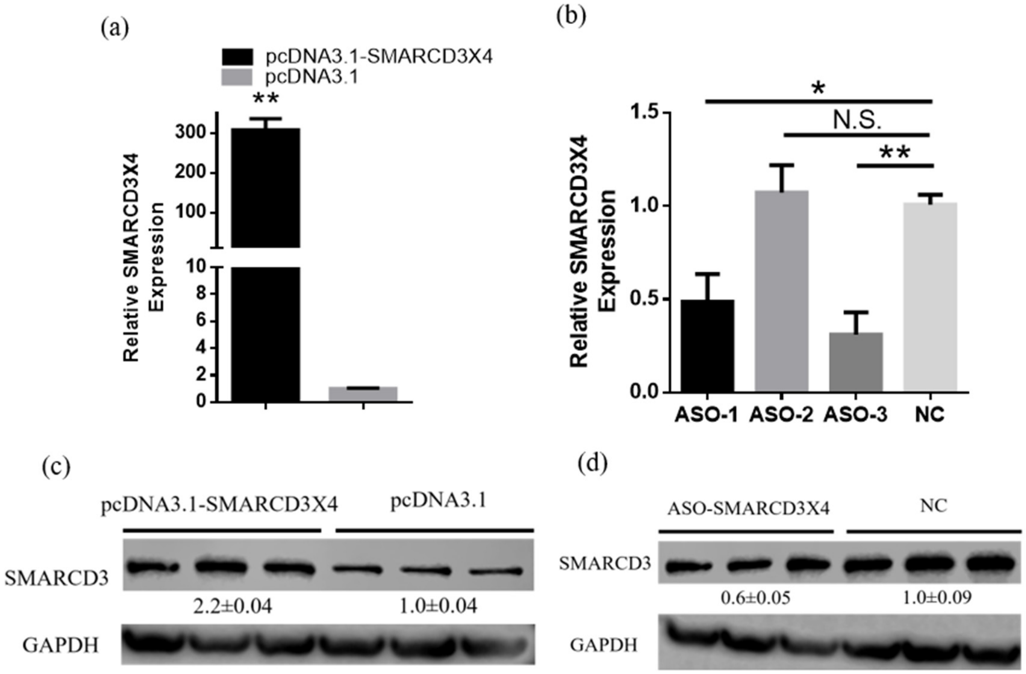

Figure S4 The fold-changes of *SMARCD3X4* in mRNA and protein levels after overexpression and knockdown. (**a**) Relative *SMARCD3X4* expression in mRNA level after introducing overexpression vector of *SMARCD3X4* into CPMs (n = 6). (**b**) Relative *SMARCD3X4* expression in mRNA level after introducing ASO of *SMARCD3X4* into CPMs (n = 6). (**c**) Relative SMARCD3X4 expression in protein level after introducing overexpression vector of *SMARCD3X4* into CPMs (n = 3). (**d**) Relative SMARCD3X4 expression in protein level after introducing ASO of *SMARCD3X4* into CPMs (n = 3). The numbers shown below the bands were folds of band intensities relative to control. Band intensities were quantified by ImageJ and normalized to GAPDH. Data are expressed as a fold change relative to the control. Results are shown as mean ± SEM. Statistical significances of differences between means were assessed using an independent sample t-test. *$p < 0.05$, **$p < 0.01$.
